# Impact of Erenumab on Migraine Disability: A Three-Month MIDAS (Migraine Disability Assessment Scale) Score Analysis at Dubai Health Facilities

**DOI:** 10.7759/cureus.67113

**Published:** 2024-08-18

**Authors:** Ali Almarzooqi, Marwan Zidan

**Affiliations:** 1 Neurology, Rashid Hospital, Dubai, ARE; 2 Statistics, Rashid Hospital, Dubai, ARE

**Keywords:** headache disorders, anti-cgrp monoclonal antibody, erenumab, migraine disability index scale (midas), migraine disorder

## Abstract

Background and aim: Migraine is a prevalent neurological disorder causing recurrent headaches that significantly impact daily life. Erenumab, a calcitonin gene-related peptide (CGRP) receptor antagonist, has emerged as a promising treatment for migraine. CGRP is thought to play a role in migraine pathophysiology, and erenumab works by blocking CGRP binding to its receptors. Erenumab has been found to be effective in reducing migraine frequency, with potential benefits for improving patient outcomes. This study investigated the impact of erenumab on migraine disability in patients treated at Dubai Health facilities. We specifically assessed changes in Migraine Disability Assessment Scale (MIDAS) scores before and after a three-month treatment period.

Methods: This retrospective analysis examined data from 26 patients diagnosed with migraine according to the established criteria. All patients received erenumab treatment for three months. MIDAS, a validated tool, was used to quantify migraine-related disability at baseline and after treatment completion. Due to potential skewness in the data distribution, the statistical analysis focused on the median change in MIDAS scores across groups based on gender and erenumab dosage. Non-parametric tests were employed to assess group differences.

Results: Erenumab treatment resulted in a median decrease of 13 points in MIDAS scores, suggesting a potential improvement in migraine disability at three months. Statistical analysis revealed no statistically significant group differences regarding MIDAS score changes between genders or erenumab dosage groups. However, trends toward improvement were observed in all subgroups.

Conclusion: While not statistically significant due to the limited sample size and the absence of a control group, these findings suggest a potential benefit of erenumab in reducing migraine disability. Future research with more extensive, controlled trials is warranted to definitively assess erenumab's effectiveness and explore potential treatment regimen variations for optimal patient outcomes.

## Introduction

Migraine is commonly characterized by severe, episodic headaches often accompanied by nausea, vomiting, and heightened sensitivity to light or sound. It is commonly regarded as a disabling neurological disorder affecting 12% to 15% of the global population [1]. Many maintenance therapies have been approved and are currently used to manage the disorder, covering a variety of proposed pathophysiological theories. However, migraine remains one of the most undertreated neurological conditions, causing afflicted patients to be disabled and unable to perform optimally [2].

One of the newer studies suggested that calcitonin gene-related peptide (CGRP) plays an important role in the pathophysiology of migraine. Studies have demonstrated that CGRP levels got elevated in the cranial circulation when migraine was induced when compared to controls. Furthermore, it has been postulated that most of the migraine treatments, such as triptans and botulinum toxin type A, indirectly lower the CGRP levels [3]. Increases in the cranial and extra-cranial circulation levels of CGRP have been shown to increase cerebral blood flow through dilation of cerebral blood vessels, increasing nociceptive signals from said blood vessels to the pain centers in the brain [4]. Other studies have demonstrated that an increase in the CGRP level is directly linked to an increase in the release of other inflammatory mediators (e.g., from mast cells), further exacerbating the “inflammatory soup” [5].

Erenumab is a monoclonal antibody that targets CGRP receptors. It is administered as a subcutaneous injectable; it is typically self-administered once the administration process has been demonstrated to the patient in a clinical setting. The drug is administered monthly. According to the reported adverse effects, the most common are tolerable by the patients, such as constipation, pain at the injection site, and upper respiratory tract infections [6]. Studies have been conducted worldwide (mainly in the United States and Europe) to measure the efficacy of erenumab; outcomes assessed in the most renowned studies, such as ARISE, STRIVE, and LIBERTY, were migraine frequency, acute migraine medication use, migraine effect on daily activity, and its effect on refractory migraine, all of which responded favorably to erenumab versus placebo [7-9].

Disability scoring has been done using different means; however, a study in 2020 looked back at the data from STRIVE and ARISE trials and applied specific and standardized disability scoring systems, one being the Migraine Disability Assessment Scale (MIDAS) questionnaire. In both studies, erenumab showed statistically significant improvement in disability scoring systems compared to the placebo [6].

Research efforts are underway globally to estimate the effect of various management strategies in reducing headache disability, including migraines. A staggering 1.04 billion people worldwide are estimated to suffer from migraine headaches, with 45.1 million years of life lived with disability (YLDs), in 2016, as revealed by a comprehensive study. This underscores our study's global relevance and urgency, designed to advance our understanding of migraine management [10].

Several studies have been conducted in the United Arab Emirates (UAE) focusing on migraine, with the most notable being conducted by Alsaadi et al. in 2022. This study measured the effectiveness of erenumab in reducing both monthly headache days (MHD) and monthly acute migraine-specific medication days (MSMD) [11]. Building on this research, our study aims to make a unique contribution by investigating the effect of erenumab on MIDAS scores at three months in patients treated at Dubai Health facilities, providing valuable insights into the local context.

After illuminating the profound impact of migraines and their global burden, particularly in terms of disability, it becomes evident why this is the primary outcome of our study. This retrospective study, conducted within the framework of the patient-reported outcome measurement system, specifically the MIDAS score, aims to compare patient outcomes in a cohort diagnosed with migraine. We analyzed the impact of erenumab on migraine disability, showing the difference before (or at baseline) and after using the medication for three months. The goal was to evaluate the effectiveness of erenumab in reducing migraine disability as measured by the MIDAS score. This study holds significant value as it is the only regional study, in our knowledge, that measures the effectiveness of erenumab in migraine disability as measured by the MIDAS score, providing crucial insights for healthcare professionals and researchers.

## Materials and methods

This retrospective study was designed to investigate the effect of erenumab (Aimovig) on the MIDAS score over a period of three months. The study included patients treated at Dubai Health facilities, ensuring a diverse and representative sample. Electronic Medical Records (EMRs) of Dubai Health facilities served as the data source for this study; the specific EMR system used was Epic EMR.

Study population

In 2023, 90 patients diagnosed with episodic and chronic migraine (CM) fulfilling the diagnostic criteria for migraine with or without aura, established in the International Classification of Headache Disorders, 3rd Edition (ICHD-3), were followed up at Dubai Health facilities and started on erenumab. Out of 90 patients, a total of 26 fulfilled the eligibility criteria. The inclusion criteria included an age range of 18-65 years; this range was chosen to avoid substantial comorbidities that might act as confounding factors, which was also double-checked while reviewing a patient’s medical record. Other inclusion criteria included treatment with erenumab for at least three months and having an EMR within the Dubai Health system. Also, their MIDAS scores should have been recorded at baseline and at the three-month follow-up.

Patients were excluded from the study if they used additional migraine prophylactic medications (other than erenumab), had incomplete or missing data on erenumab treatment or MIDAS scores, or were pregnant or breastfeeding women.

Data collection

If patients are to undergo anti-CGRP therapy for insurance coverage purposes, their medical record numbers are registered and divided according to the specific therapy they are using. The patients using erenumab were chosen from the registry and reviewed to meet the inclusion and exclusion criteria. In the registry, 90 patients used erenumab in 2023, but only 26, all UAE nationals, met the eligibility criteria (inclusion and exclusion).

Ethical considerations

This study was conducted with an ethical approval from the Dubai Scientific Research Ethics Committee (DSREC) on January 2, 2023 (reference number DSREC/RRP/2022/33) to conduct the study on migraine patients during the year 2023, where the study followed the anonymization protocol of not revealing the patient's identity, including the medical record number, except to the principal investigator. As such, once patients’ data were assimilated, the medical record numbers were changed to randomized single- or double-digit numbers.

Statistical analysis

Descriptive analyses were done that summarized the patient’s demographic data, MIDAS score, and erenumab dosage. The mean MIDAS difference was then tested for the correlation between age and the time frame by two MIDAS scores (i.e., at baseline and at the three-month follow-up). The mean MIDAS differences underwent a normality test, and based on that, the data was further analyzed using a paired-sample t-test to assess differences between the mean MIDAS difference in gender and different doses (70 vs. 140 mg). Statistical analyses were performed using IBM SPSS Statistics, version 28 (IBM Corp., Armonk, NY) [12].

## Results

**Figure 1:**
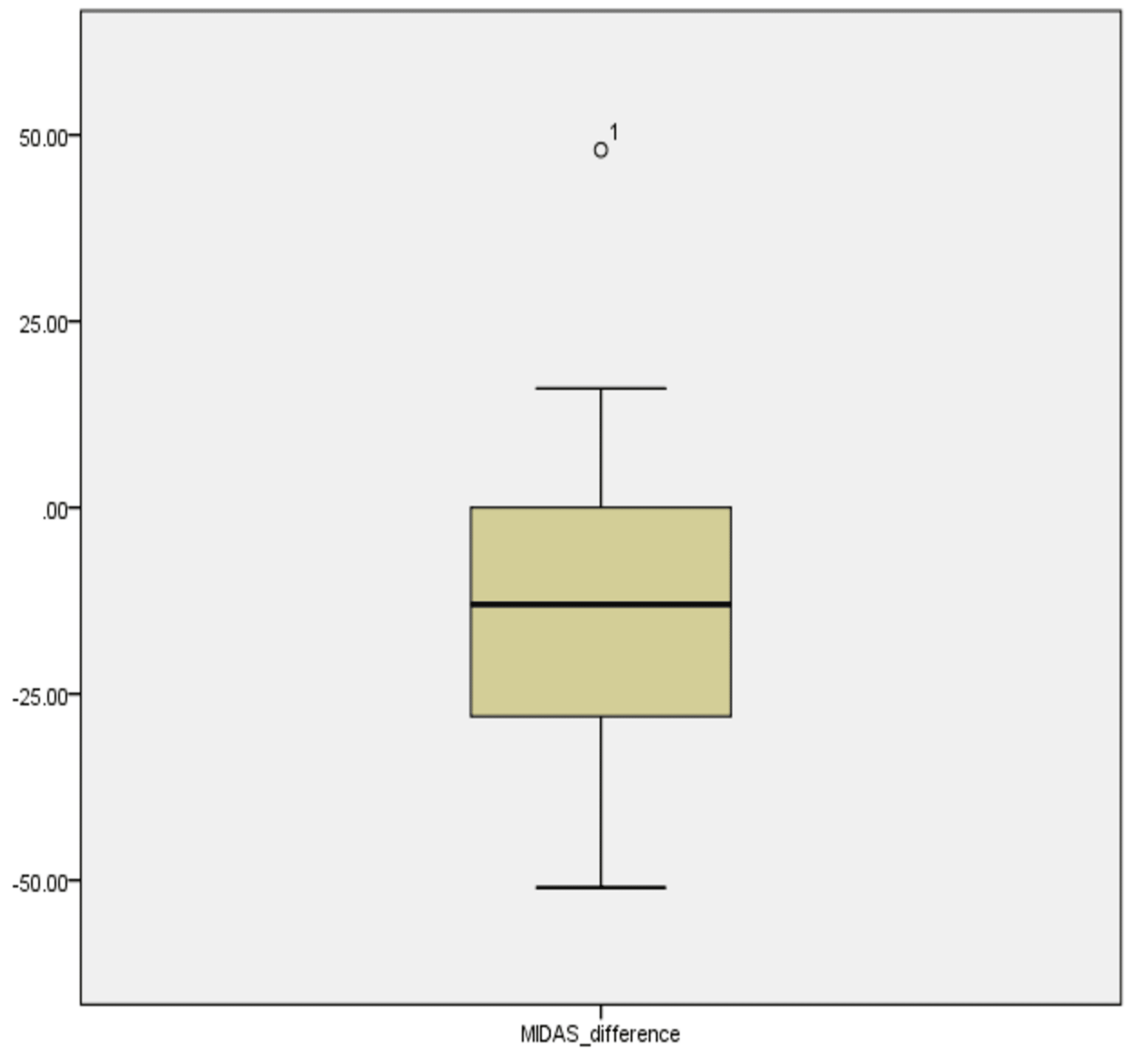
Boxplot reflecting the median MIDAS differences, pre- and post-treatment This descriptive boxplot demonstrates the MIDAS score change (between baseline and three months) for migraine patients treated with erenumab. The boxes represent the interquartile range (IQR), with the median score depicted by the horizontal line within each box (-13). Whiskers extend to the most extreme data points that are not considered outliers. MIDAS, Migraine Disability Assessment Scale

The data for the 26 patients was analyzed to clearly understand the MIDAS scores at baseline and the three-month follow-up; this was reported as a MIDAS difference. There were 18 females and 8 males who met the eligibility criteria, with similar numbers seen in the users of the erenumab doses; 18 patients were on 70 mg, while only 8 patients were on 140 mg of erenumab. The descriptive Table 1 provides an insight into the mean MIDAS difference score between baseline and at the three-month follow-up across all patients, which was -11.58. At the same time, Figure 1 delineates the median MIDAS difference of -13. Age (p = 0.899) and time frame between two MIDAS scores (p = 0.696) did not correlate statistically with the MIDAS difference score.

**Table 1:** Descriptive analysis of the collected results Erenumab treatment resulted in an average decrease (mean, -11.58) in MIDAS difference scores, indicating a potential improvement in migraine disability at the three-month follow-up. There was a moderate spread in individual responses, with some participants experiencing a more significant decrease in migraine disability compared to others. Age and the time frame between MIDAS assessments showed no statistically significant correlations with the change in migraine disability scores. MIDAS, Migraine Disability Assessment Scale

Variables	Value
Mean MIDAS difference across all data	-11.5769
Number of female patients	18
Number of male patients	8
Number of patients receiving 70 mg erenumab	18
Number of patients receiving 140 mg erenumab	8

**Figure 2:**
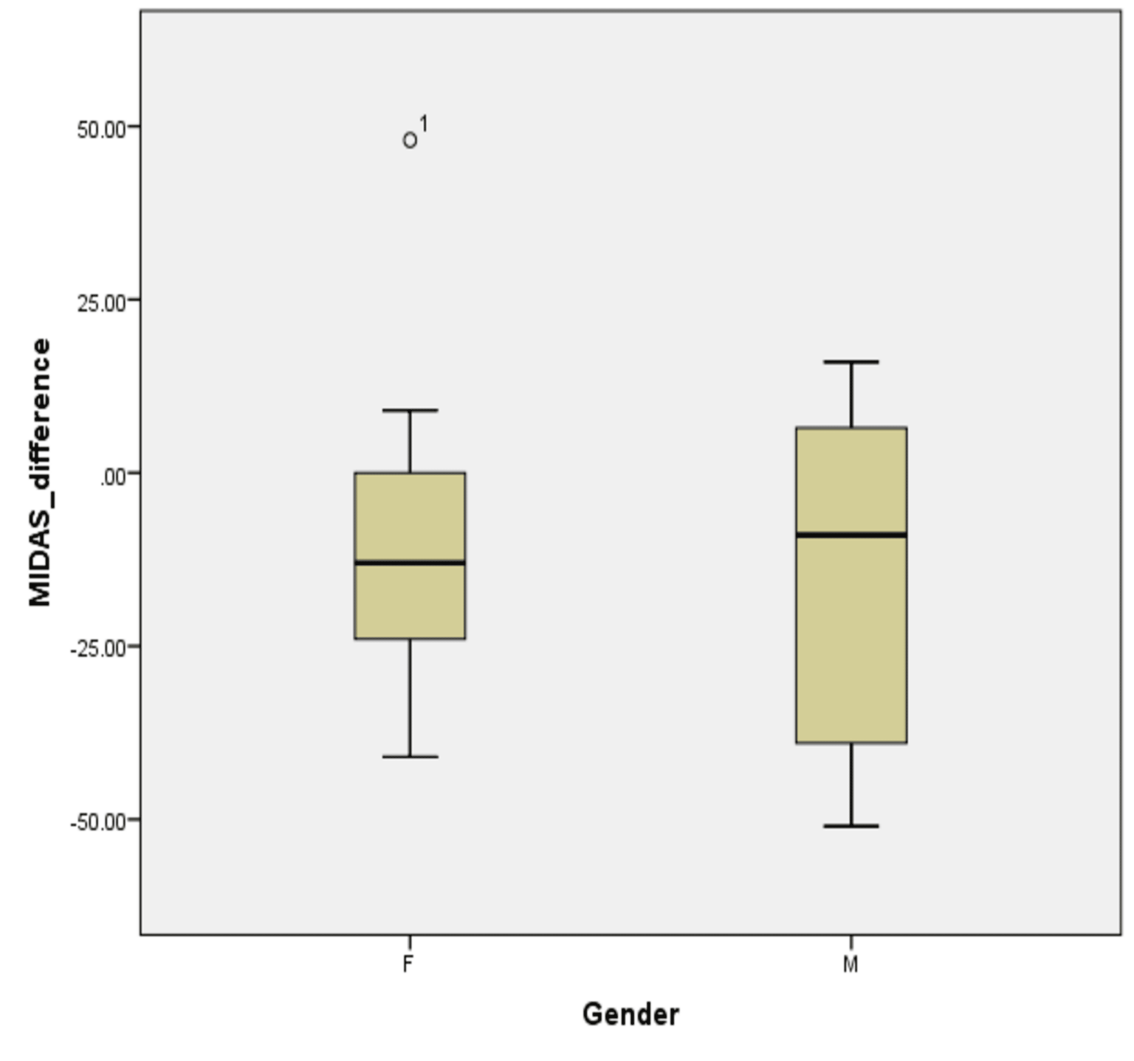
Boxplot reflecting the median MIDAS difference in relation to genders This boxplot demonstrates the change in the MIDAS score (difference between baseline and three months) for migraine patients stratified by gender and treated with erenumab. The boxes represent the interquartile range, with the median score depicted by the horizontal line within each box (M= -9, F= -13). MIDAS, Migraine Disability Assessment Scale; M, male; F, female

Further cohort analyses were conducted by grouping patients based on gender. As illustrated in Figure 2, the median MIDAS differences reflect the difference between the males and females, -9 and -13, respectively. The mean differences in MIDAS scores across different genders showed a p-value of 0.105 for females and 0.442 for males in the Shapiro-Wilk normality test; the data is not inconsistent with a normal distribution as seen in Table 2. The initial descriptive values in Table 3 reflect the mean MIDAS differences between the two genders, with males having a mean of -14.75 and females -10.17.

**Table 2:** Normality test results for gender and dosage variables This table presents the p-values from the Shapiro-Wilk normality tests for the gender and dosage variables in the study. These tests determine if the data distribution deviates from a normal distribution, with higher p-values indicating a greater likelihood that the data is normally distributed.

Test	Variable	p-value
Shapiro-Wilk (normality)	Gender (female)	0.105
	Gender (male)	0.442
	Dosage (70 mg)	0.555
	Dosage (140 mg)	0.599

**Table 3:** Comparative analysis of MIDAS score differences by gender and dosage This table provides a comparative analysis of the median and mean MIDAS score differences for migraine patients treated with erenumab, stratified by gender and dosage. The independent samples t-test and F-test (Levene's test) results are included to evaluate the statistical significance of differences in MIDAS scores. The t-values and p-values from the t-test assess the mean difference significance, while the F-values and p-values from Levene's test check the equality of variances. MIDAS, Migraine Disability Assessment Scale

Variable	Median	Mean MIDAS difference	t-values	p-value (t-test)	F-value	p-value (F-test)
Gender (female)	-13	-10.17	-0.493	0.626	1.461	0.239
Gender (male)	-9	-14.75				
Dosage (70 mg)	-10	-9.61	0.691	0.496	0.004	0.947
Dosage (140 mg)	-17.5	-16.00				

The mean difference between the two genders was -4.58; an independent samples t-test was done. The p-value for Levene's test was 0.239 suggesting that the variances of the two groups are likely equal. The p-value for the t-test was 0.626 as seen in Table 3.

**Figure 3:**
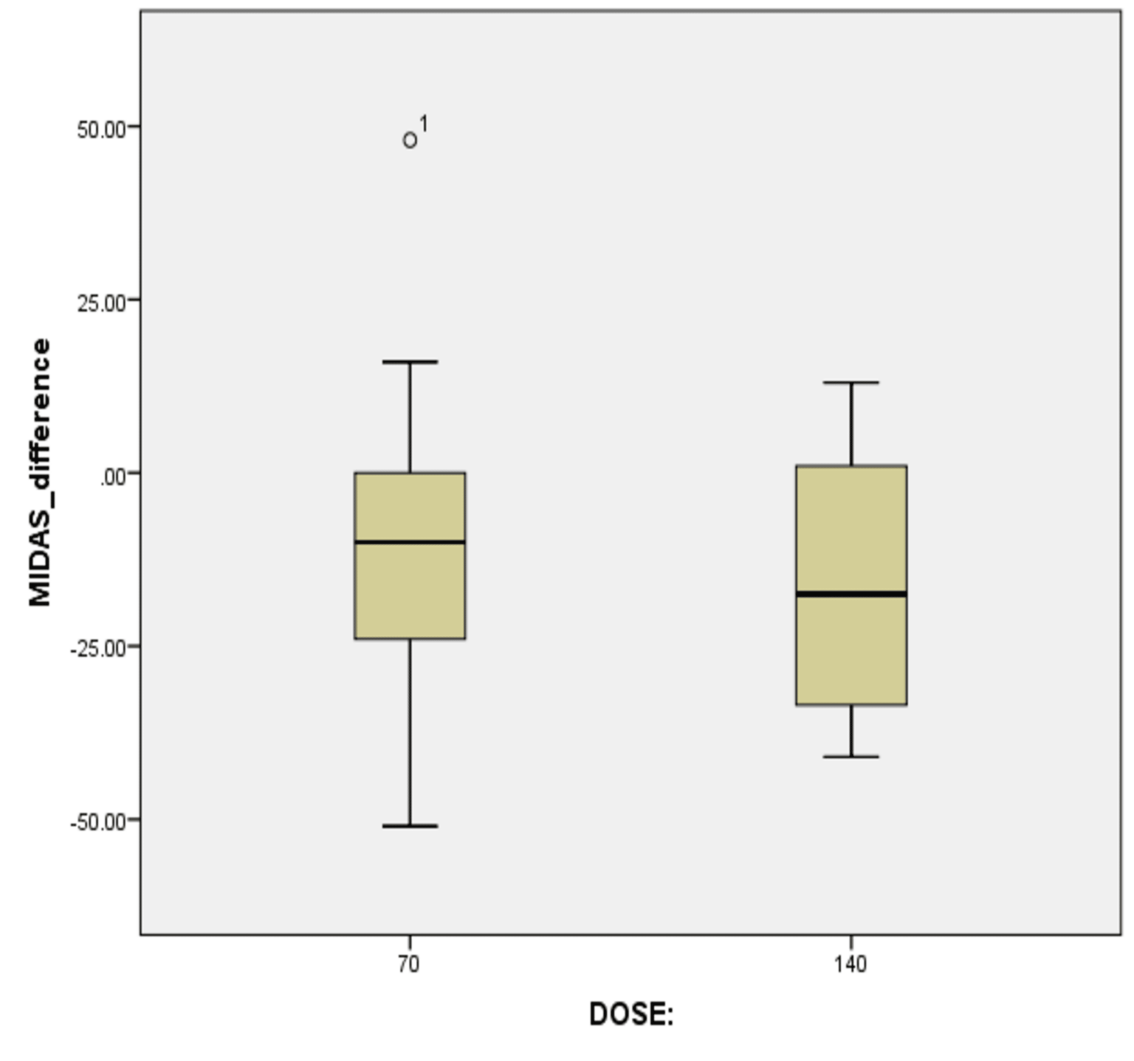
Boxplot reflecting the median MIDAS differences between different erenumab dosages used This boxplot demonstrates the change in MIDAS scores (difference between baseline and three months) for patients with migraine treated with two different dosages of erenumab. Notably, the median change in the MIDAS difference appears to be more significant in the group receiving the higher dose of erenumab (140 mg), i.e., -17.5 compared to the group receiving the lower dose (70 mg), i.e., -10. MIDAS, Migraine Disability Assessment Scale

Finally, the cohort data was grouped based on dosage differences. The average MIDAS difference between 70 mg and 140 mg doses was -9.6 and -16 respectively, while the medians were -10 and -17.5 as demonstrated in Figure 3. Similarly, the means were subjected to the Shapiro-Wilk normality test, showing a p-value of 0.555 for 70 mg, while for 140 mg, the p-value was 0.599.

An independent samples t-test was conducted to evaluate the impact of erenumab dosage on MIDAS scores. Levene's test confirmed the homogeneity of variances between the 70 mg and 140 mg erenumab groups (F = 0.004, p = 0.947). The t-test showed no statistically significant difference in the mean MIDAS score change between the two dosage groups (t = 0.691, p = 0.496). While the mean difference in MIDAS scores favored the 140 mg dose by 6.39 points, this difference did not reach statistical significance.

## Discussion

A migraine is a disabling headache that affects daily functionality of the affected individual, ultimately reducing productivity and leading to psychological effects [13]. This study investigated the impact of erenumab on migraine disability, as measured by the MIDAS score, in a group of migraine patients. The patients received either a 70 mg or 140 mg dose of erenumab for three months. The results did not reveal statistically significant differences in MIDAS scores between baseline and at the three-month follow-up, genders, or between the 70 mg and 140 mg dosage groups. The data showed a trend toward improvement in migraine disability. The mean and median MIDAS difference scores were negative in all groups mentioned above, indicating a decrease in disability at the three-month follow-up. It is important to note that the data on MIDAS score changes exhibited a potentially skewed distribution. Therefore, the median, rather than the mean, is presented as a more representative measure of central tendency in the figures and descriptive analysis.

Our findings are in line with a previous research that demonstrated the efficacy of erenumab in reducing migraine-related disability. In a phase 3 randomized, double-blind, placebo-controlled trial, Dodick et al. evaluated the efficacy and safety of erenumab for preventing episodic migraine (EM) in 955 patients. The study reported that erenumab significantly reduced the number of migraine days per month, with patients receiving 70 and 140 mg experiencing reductions of 3.2 and 3.7 days, respectively [7]. Similarly, Goadsby et al. conducted a randomized, double-blind, placebo-controlled trial involving 667 patients with CM. They found that erenumab significantly reduced monthly migraine days and improved quality of life. Patients treated with 70 and 140 mg doses reported reductions of 6.6 and 6.4 days per month, respectively [8]. Although our study did not achieve statistical significance, the observed trend toward a decrease in MIDAS scores aligns with the reductions in migraine days and improvements in patient outcomes reported in these more extensive, controlled trials [7-8]. These consistent trends underscore the potential utility of erenumab in clinical practice, and even though our study was conducted with a relatively small sample, our findings support the need for further investigation through larger, well-designed studies to confirm the benefits of erenumab in reducing migraine-related disability.

A study done in multiple centers throughout the UAE by Alsaadi et al. in 2022 investigated the effectiveness and tolerability of erenumab for migraine prevention in the UAE [11]. The study assessed its impact by measuring MHD and monthly acute MSMD reductions. Erenumab was found to be effective in reducing these for both CM and EM patients. The study enrolled 166 patients and observed clinically meaningful reductions in MHD and MSMD at all assessed time points, with most patients achieving at least a 50% reduction from baseline. Erenumab was also well-tolerated, and no new safety concerns were identified. These findings suggest that erenumab is a safe and effective treatment option for migraine prevention in patients with CM or EM in the UAE [11].

Several factors might explain the lack of statistically significant findings. With a larger sample size, the study might have been able to identify subtle effects, as suggested by the observed trend toward improvement [13]. Additionally, a more extended follow-up period beyond three months might be needed to capture the full impact of erenumab treatment, considering some studies reported delayed improvements [14]. Finally, the heterogeneity of the study population, which likely included a variety of migraine subtypes and severities, could have masked the overall treatment effect [15]. Notably, the absence of statistical significance does not necessarily equate to a lack of treatment effect.

In the previous researches on erenumab's impact on MIDAS scores, the results were inconclusive, as the studies yielded conflicting results [14-15]. Our findings align with some previous research suggesting a trend toward improvement, highlighting the need for further investigation with larger, more controlled designs to assess the effectiveness of erenumab on migraine disability definitively, as measured by MIDAS scores.

Limitations

This study also has limitations that need to be considered. The small sample size might have limited the study's ability to detect statistically significant differences. Additionally, the single-arm, pre-post design without a control group limits the ability to establish a causal relationship between erenumab and the observed changes in MIDAS scores. Furthermore, the unequal distribution of participants across genders (more females than males) and treatment dosages (more in the 70 mg group compared to the 140 mg) might limit the generalizability of findings regarding gender differences and treatment effects based on dosage.

Future research is needed to explore the effectiveness of erenumab on migraine disability in more detail. Conducting larger, randomized controlled trials with a control group receiving a placebo would provide more substantial evidence regarding erenumab's efficacy; this could include investigating a broader patient population or catchment area. Investigating the long-term effects of erenumab on migraine disability beyond three months could also be beneficial. Finally, examining the impact of erenumab on MIDAS scores in different subgroups based on migraine severity, subtype, gender, and dosage could provide valuable insights.

## Conclusions

While this study's findings suggest a potential benefit of erenumab in reducing migraine disability, further research is necessary to confirm its efficacy. Larger, randomized controlled trials with a control group and extended follow-up periods will provide more robust evidence regarding erenumab's impact on migraine disability as measured by MIDAS scores. Additionally, future studies should explore the effects of erenumab across different subgroups, including variations in migraine severity, subtypes, and compare it with other prophylactic migraine medications, to identify which patients benefit most from this treatment. Investigating the long-term effects of erenumab beyond three months and its impact on quality of life and daily functioning is also crucial.
